# Validity of data collected in BIOREG, the Austrian register for biological treatment in rheumatology: current practice of bDMARD therapy in rheumatoid arthritis in Austria

**DOI:** 10.1186/s12891-016-1207-4

**Published:** 2016-08-22

**Authors:** Bernhard Rintelen, Jochen Zwerina, Manfred Herold, Franz Singer, Johann Hitzelhammer, Wolfgang Halder, Gabriela Eichbauer-Sturm, Rudolf Puchner, Miriam Stetter, Burkhard F. Leeb, on behalf of the BIOREG investigator group, Elke Böttcher, Lothar Boso, Hans Peter Brezinschek, Attila Dunky, Gabriele Eberl, Vera Ferincz, Markus Gaugg, Gudrun Lechner, Harsono T. H. Mai, Erich Mur, Monika Mustak-Blagusz, Valerie Nell-Duxneuner, Andrea Österbauer, Peter Petera, Judith Sautner, Martin Steindl, Otto Stummer, Gerhard Teich, Wolfgang Thoma, Anna Totzauer, Andrea Trenkler, Armin Vesenmayer, Jeanette Wolf

**Affiliations:** 1 Lower Austrian State Hospital Stockerau, 2nd Department for internal medicine, Lower Austrian Center for Rheumatology, Landstrasse 18, Stockerau, 2000 Austria; 2 Karl Landsteiner Institute for Clinical Rheumatology, Landstrasse 18, Stockerau, 2000 Austria; 3 Ludwig Boltzmann Institute of Osteology at the Hanusch Hospital of WGKK and AUVA Trauma Centre Meidling, 1st Medical Department, Hanusch Hospital Vienna, Heinrich-Collinstrasse 30, Vienna, 1140 Austria; 4 Medical University of Innsbruck, Anichstrasse 35, Innsbruck, 6020 Austria; 5 BIOREG, Schloßhoferstrasse 4/4/12, Vienna, 1221 Austria; 6 Health Center Vienna Mariahilf, Wiener Gebietskrankenkasse, Mariahilfer Strasse 85-87, Vienna, 1060 Austria; 7 Tyrolean State Hospital Hochzirl, Hochzirl, 6170 Austria; 8 Office based rheumatologist, Freistätter Strasse 16, Linz, 4040 Austria; 9 Office based rheumatologist, Freiung 19, Wels, 4070 Austria; 10 Department for Internal Medicine, Lower Austrian State Hospital Amstetten, Krankenhausstrasse 21, Amstetten, 3300 Austria; 11 Medical University of Graz, Auenbruggerplatz 2, Graz, 8010 Austria

## Abstract

**Background:**

The purpose of the present study was to check the validity of data collected in BIOREG, the Austrian register for biological treatment in rheumatology, and to elucidate eventual differences with respect to disease activity (DA) in patients with rheumatoid arthritis (RA) on established biological DMARDs (bDMARDs) before inclusion into the register (EST) and beginners at the time point of inclusion (NEW) after 1 year of treatment.

**Methods:**

RA patients with a complete follow-up of 1 year in BIOREG were divided into EST and NEW and compared with respect to DA, remission rates, concomitant synthetic DMARDs (csDMARDs) and glucocorticoid therapy (GC) at baseline and after 1-year follow-up. Safety concerns are listed. Descriptive statistics are applied.

**Results:**

For 346 RA patients (284 EST, 62 NEW) out of 970 RA patients included into BIOREG, a full data set for a 1-year follow-up was available. No differences in DA were observed after 1 year as expressed by DAS28 or RADAI-5, and small differences as expressed by remission rates according to DAS28, RADAI-5 or Boolean criteria (namely approximately 1/2, 1/3 to 1/4 and 1/4 to 1/5 of the patients respectively). Sixty-four adverse events (AEs) were noted in 56 (20 %) of EST and 20 in 19 (31 %) of NEW patients. Malignancy occurred in four patients. After 1 year, 48 % of EST patients but only 16 % of NEW patients were on bDMARD monotherapy.

**Conclusion:**

Regarding DA, the date collected in BIOREG appeared to be valid. After 1 year of bDMARD therapy, all patients, whether EST or NEW, achieved a similar level of DA. AEs occurred more frequently during the early phase of bDMARD treatment. Austrian rheumatologists initiate bDMARD therapy in patients with lower disease levels than in other European countries, leading to high remission rates.

## Background

BIOREG, the Austrian register for patients with chronic rheumatic diseases treated with biological agents, was established in 2010 [1]. Up to the time of the data report in October 2015, 970 patients suffering from rheumatoid arthritis (RA), 276 from psoriatic arthritis, 407 from spondylarthritis and 51 from other chronic rheumatic diseases such as systemic lupus erythematodes, juvenile arthritis, chronic inflammatory bowel disease and Still disease were included into the registry. The primary goal of the register is to document safety concerns, as well as disease activity (DA) and changes of therapy, and to collect socioeconomic data. Twenty Austrian centres currently take part in BIOREG (eight rheumatologists in private offices and 12 specialized rheumatology outpatient clinics in public or private hospitals in all nine federal states of Austria). BIOREG is a member of the European Network of Centres for Pharmacoepidemiology and Pharmacovigilance (ENEPP), is in close partnership with the Austrian Society for Rheumatology and Rehabilitation (ÖGR) and is supported by an unrestricted industrial grant. Data analysis is performed annually in September.

The intention of this study was to check the validity of the collected data, as well as to get insights into the “real-world” use of bDMARDs (biologic Disease Modifying Antirheumatic Drugs) in Austria. Therefore, we compared patients included into BIOREG when starting bDMARD therapy (NEW) with those on established bDMARD therapy (EST) at the time point of inclusion. Disease activity in general, remission rates, concomitant conventional synthetic DMARDs (csDMARDs), concomitant glucocorticoid therapy (GC) and adverse events were analysed at the time point of inclusion after 1 year. We hypothesized that, emanating from difference at inclusion, DA should be comparable in EST and NEW after 1 year of inclusion in BIOREG. Furthermore, for plausibility, we compared our results with other registers, e.g. the German register RABBIT [2].

## Methods

To be included into this evaluation, it was a prerequisite that a full data set be available for the respective patients at the time point of inclusion and after 1 year of observation. We defined NEW patients as starting on a bDMARD at the time point of registration in BIOREG (+/- 30 days); all other patients were regarded as EST patients.

In all patients, the number of tender and swollen joints (TJ, SJ) based on the 28-joint count, the erythrocyte sedimentation rate in the first hour (ESR) and the patient’s global assessment of the disease on a 100 mm visual analogue scale (VASGH), as well as the physician’s global assessment of disease activity on a 100 mm visual analogue scale (VASPH), were available. The DAS28-ESR was calculated [3], and additionally patient-related outcomes, such as the RADAI-5 [4] and the health assessment questionnaire disability index (HAQ-DI), were documented [5].

With respect to efficacy, remission rates according to the DAS28-ESR [6], the Boolean criteria (BC) on the basis of the 2011 ACR/EULAR remission criteria [7] and remission as defined by the RADAI-5 disease activity categories [8] after 1 year were compared. In addition, concomitant therapies with csDMARDs as well as GC were evaluated, along with the percentage of patients receiving biological mono-therapy. With respect to tolerability, adverse events (AEs) and co-morbidities during the 1-year treatment period with bDMARDs were compared in both groups, analysed in relation to all patients with missing data. Primarily descriptive statistics were applied. Where appropriate, median values and the 25th to 75th interquartile range or percentages were calculated. The deadline for inclusion into this study was the end of August 2015 (report dated October 2015).

All patients gave their informed consent before inclusion into BIOREG, which is treated as a prospective observational study. Collection of data is completely anonymous, using printed case report forms that are collected in an office for computerizing and analysing [1]. The central computer is not connected to the World Wide Web, to avoid data abuse. Data are collected six-monthly (+/- 3 months). The ethical committee of Lower Austria has approved the study design of BIOREG (Reference number GS4-EK-085-2009).

## Results

The records of 346 patients complied with the prerequisite of a complete data set, as described, at inclusion and after 1 year of follow-up (36 % of all in BIOREG included RA patients, EST *N* = 284, NEW *N* = 62).

As shown in the demographic data (Table 1), EST patients show a 5-year longer median disease duration than NEW patients; they are on bDMARD therapy for a median duration of 3.2 years; no age-related differences are found; rheumatoid factor and/or ACPA are found positive in 68 % of the EST patients and in 55 % of the NEW patients; and co-morbidities are recorded in 64 % of the EST patients and in 73 % of the NEW patients, respectively. In EST patients, these disorders most frequently comprise cardiovascular diseases, which account for 27 %, followed by dyslipidemia in 16 %, osteoporosis in 14 %, depression in 10 % and diabetes in 9 %; the respective percentages for NEW patients are 42, 16, 10, 15 and 10 %. As expected proof of bDMARD treatment efficacy, EST patients have milder disease at inclusion than NEW patients, as expressed by the DAS28 and the RADAI-5 (mild versus moderate disease activity according to both scores), as well as by the HAQ-DI.

**Table 1:** Demographics of RA patients included in BIOREG at baseline (if not otherwise indicated, median (25th and 75th percentile))

	All pts	EST	New
n	970	284	62
Age (years)	58.0 (50.0; 68.0)	59.0 (48.0, 67.0)	58.0 (47.0, 70.0)
Disease duration (years)	8.0 (4.0, 14.0)	9.00 (5.0, 14.0)	4.00 (1.00, 9.00)
Gender % female	77.3	78.4	77.4
RF pos %	69.3 (*n* = 894)	68.0 (*n* = 264)	54.8 (*n* = 59)
ACPA pos %	47.6 (*n* = 633)	50.0 (*n* = 193)	37.1 (*n* = 46)
Duration of bDMARD treatment prior inclusion (years)	1.99 (0.22, 5.18)	3.22 (1.53, 6.02)	na
DAS28	3.26 (2.24, 4.20)	2.67 (1.99, 3.70)	4.46 (3.77, 5.35)
RADAI-5	3.2 (1.6, 4.8)	2.6 (1.2, 4.4)	4.4 (3.2, 6.2)
HAQ-DI	0.75 (0.25, 1.25)	0.63 (0.13, 1.19)	1.00 (0.63, 1.38)
GC %	35.9	25.0	66.1
csDMARD %	64.6	56.0	83.9

*Abbreviations*: *ACPA* Anti Citrullinated Peptide Antibodies, *bDMARD* biological DMARD, *csDMARD* concomitant conventional synthetic DMARD, *DAS28* Disease Activity Score using ESR out of 28 joints, *ESR* erythrocyte sedimentation rate, *EST* pts with established bDMARD treatment before inclusion in BIOREG of whom a full data set as described is viable, *GC* concomitant glucocorticoid treatment, *HAQ*-*DI* Health Assessment Questionnaire Disability Index, *N* number of patients, *NA* not applicable, *NEW* pts included in BIOREG with start of biologic treatment ± 30 days of whom a full data set as described is viable, *PTS* patients, *RA* rheumatoid arthritis, *RADAI*-*5* Rheumatoid Arthritis Disease Activity Index-5, *RF* Rheumatoid Factor

After 1 year, on the group level, both EST and NEW patients can be categorized as being in remission according to DAS28 and in a mild disease activity stage according to the RADAI-5 (Table 2). Accordingly, NEW patients experienced a good EULAR response [9] and a significant response according to the RADAI-5 [10]. There was no significant change on either score in EST patients. Regarding remission as measured by the DAS28, the RADAI-5 and the BC, after the 1-year observation period, more than half of the patients were found in DAS28, approximately 27–36 % in RADAI-5 and 21–26 % in remission according to the BC, respectively (Table 3). Additionally, no differences in HAQ-DI-scores were observed (Table 2).

**Table 2:** Changes of disease activity and HAQ-DI in EST and NEW patients (median (25th and 75th percentile))

	EST (*n* = 284)		NEW (*n* = 62)	
	baseline	after 1 year	baseline	after 1 year
DAS28	2.67 (1.99, 3.70)	2.58 (1.80, 3.45)	4.46 (3.77, 5.35)	2.43 (1.54, 3.57)
DAS28 Diff		0.18 (-0.47, 0.87)		1.92 (0.65, 2.99)
RADAI-5	2.6 (1.2, 4.4)	2.2 (1.0, 4.0)	4.4 (3.2, 6.2)	2.4 (1.2, 3.8)
RADAI-5 Diff		0.2 (-0.5, 1.2)		2.0 (0.6, 3.4)
HAQ	0.63 (0.13, 1.19)	0.63 (0.13, 1.13)	1.00 (0.63, 1.38)	0.50 (0.13, 1.25)
HAQ Diff		0.00 (-0.13, 0.25)		0.38 (0.00, 0.63)

*Abbreviations*: *DAS28* Disease Activity Score using ESR out of 28 joints, *DIFF* Difference to baseline after 1 year follow up, *ESR* Erythrocyte Sedimentation Rate, *EST* pts with established bDMARD treatment before inclusion in BIOREG of whom a full data set as described is viable, *HAQ*-*DI* Health Assessment Questionnaire Disability Index, *N* number of pts, *NEW* pts included in BIOREG with start of biologic treatment ± 30 days of whom a full data set as described is viable, *PTS* patients, *RADAI*-*5* Rheumatoid Arthritis Disease Activity Index-5

**Table 3:** Percentages of remission at baseline and 1-year follow-up and percentages of concomitant csDMARD and GC therapy in EST and NEW patients

	EST *n* = 284		NEW *n* = 62	
	baseline	after 1 year follow up	baseline	after 1 year follow up
DAS28 remission %	47.2	51.1	1.6	53.2
RADAI-5 remission %	31.3	36.3	1.6	27.4
BC remisson %	21.1	26.1	1.6	21.0
GC%	25.0	27.8	66.1	48.4
csDMARD %	56.0	51.8	83.9	67.7
bDMARD %	100.0	94.0	na	87.1

*Abbreviations*: *BC* Boolean criteria, *bDMARD* biologic DMARD, *csDMARD* conventional synthetic DMARD, *DAS28* Disease Activity Score using ESR out of 28 joints, *ESR* Erythrocyte sedimentation rate, *EST* pts with established bDMARD treatment before inclusion in BIOREG of whom a full data set as described is viable, *GC* glucocorticoid; na not applicable, *NEW* pts included in BIOREG with start of biologic treatment ± 30 days; pts patients, *RADAI*-*5* Rheumatoid Arthritis Disease Activity Index-5

With respect to concomitant treatment at the time point of inclusion into BIOREG, a smaller percentage of patients receiving csDMARDs and GC in the EST group were found compared to the NEW patients (Table 3), as expected. Forty-four percent of the EST patients took no csDMARDs, while only 16 % of the NEW patients were on monotherapy with a biological drug. In EST patients, the most frequently applied csDMARD was Methotrexate (MTX) in 47 %, followed by Leflunomide (LEF) in 6 %, Sulphasalazine (SSZ) in 2 %, and in 2 % combinations of these three csDMARDs. In the NEW group, the numbers were 63 % MTX, 16 % LEF, 2 % SSZ and 2 % combination, respectively.

After 1 year of treatment, csDMARD therapy was stopped in 4 % of the EST patients and in 16 % of the NEW patients. At the 1-year follow-up visit, 25 patients (7.2 % of all patients; EST 17 (6 %), NEW 8 (13 %)) had no concomitant bDMARD considering an accepted drug-free period of two recommended application intervals. Causes for decanting bDMARDs in EST patients were remission according DAS28 and/or CDAI and/or RADAI-5, the patient’s wish and fear of AEs. In the eight NEW patients, the reasons were intolerance, bDMARD ineffectivity, the patient’s wish and, in one case, newly diagnosed prostate cancer. Just 17 of all these patients were on csDMARD therapy (9 EST, 8 NEW).

With respect to concomitant GCs, considerable differences between the two groups persisted. Whereas in the EST group 25 % of the patients were on GCs at inclusion and 28 % after 1 year, the frequency of GC users in NEW patients was notably greater, namely 66 % at entry and 48 % after 1 year.

After 1 year, 64 AEs were observed in 56 EST patients (20 % of all EST patients) and 20 AEs in 19 NEW patients (31 %) (Table 4); in all available records after 1 year of observation (*n* = 511), AEs appeared to occur in a total of 27 % of patients. Analysis of the recorded AEs of EST and NEW patients in comparison to all reported AEs in RA patients after 1 year in BIOREG, showed that infections in several locations were most frequent in both groups and comparable to all reported AEs (approximately 40 %). Mostly upper and lower respiratory tract infections (40 % of reported infections) occurred, followed by common colds (17 %), urinary tract infections (12 %), herpetic infections (12 %), encapsulated infections such as septic arthritis or abscess (7 %), gastrointestinal infections (7 %) and others (5 %). Local drug reactions were much more common in NEW than in EST patients. No opportunistic infection was reported.

**Table 4:** Adverse events during first year of observation (+/- 3 months). Adverse events reported fewer than three times are not listed, except that there were at least three reported adverse events in one group of patients. (Number of reported cases (in N of patients), % of all reported adverse events). The last two columns describe adverse events recorded in all patients included in BIOREG RA-group with completed 1-year observation (*n* = 511)

	EST (in 19.7 % of pts)		NEW (in 30.7 % of pts)		All RA pts in BIOREG with one year of observation (in 26.8 % of pts)	
All	64 (56)	100 %	20 (19)	100 %	153 (137)	100 %
SAE	31 (23)	48.4 %	4 (4)	20.0 %	72 (58)	47.1 %
Infections	25 (23)	39.1 %	8 (8)	40.0 %	60 (56)	39.2 %
Ulcerative skin lesions	4 (4)	6.3 %	1 (1)	5.0 %	6 (6)	3.9 %
Malignant diseases	3 (3) ^a^	4.7 %	1 (1) ^b^	5.0 %	6 (6) ^c^	3.9 %
Ophthalmic diseases	3 (3)	4.7 %	0		7 (7)	4.6 %
Thrombotic diseases	3 (3)	4.7 %	1 (1)	5.0 %	4 (4)	2.6 %
Intolerance of biologic drug	1 (1)	1.6 %	3 (3)	15.0 %	9 (9)	5.9 %
Vertigo	3 (2)	4.7 %	0		3 (2)	2.0 %
Others	22(17)	34.4 %	6 (6)	30.0 %	58 (55)	37.9 %

*Abbreviations*: *EST* pts with established bDMARD treatment before inclusion in BIOREG of whom a full data set as described is viable, *NEW* pts included in BIOREG with start of biologic treatment ± 30 days of whom a full data set as described is viable, *PTS* patients, *RA* rheumatoid arthritis, *SAE* serious adverse event

^a^ relapse of breast cancer, B-cell lymphoma, thyroid cancer

^b^ prostate cancer

^c^ as listed plus colorectal cancer and lung cancer

The proportion of serious adverse events (SAEs) was higher in EST than in NEW patients, approximately 50 % of all reported AEs in EST and 20 % in NEW patients, respectively. Fifty-four percent of the SAEs in EST patients and 25 % in NEW patients were infections; malignant diseases occurred in four patients (three in EST patients: relapse of breast cancer, B-cell lymphoma and thyroid cancer; one in NEW patients: prostate cancer). Another two reports of malignant diseases were detected in all viable records of RA patients after 1 year of observation (one case of colorectal cancer and one of lung cancer). Thus, in summary, neither a higher occurrence rate nor organ or tumor specificity of malignancies was revealed. No fatal casualties were reported.

## Discussion

As expected, treatment of RA patients with bDMARDs proved to be effective in EST and NEW patients. NEW patients, in whom bDMARD therapy was started in moderate disease activity, achieved remission or low disease activity according to the DAS28 and the RADAI-5 after 1 year of observation, while EST patients remained stable in remission or low disease activity. After 1 year of observation, both EST and NEW patients achieved comparable DA, supporting the validity of the BIOREG data.

Remarkably high percentages of patients in remission were observed, not only according to the DAS28 (more than 50 % in both groups) or to the RADAI-5 (approximately 27 % in EST—and 36 % in NEW patients), but also according to the BC (26 % of the patients in the EST group, 21 % in the NEW group). The varying rates of patients in remission, depending upon the definition applied, are well known [11]; however, other reports dealing with remission rates do not show as high rates as presented here [12, 13]. One plausible reason for this may be that biological treatment in Austria is initiated in patients with milder disease than in other countries [14]. Better outcomes in milder than higher diseased patients are underlined by a recent study conducted in a five-centre Norwegian register [15], as well as in the German register RABBIT, which recorded a 30 % DAS28 remission rate when bDMARD therapy was started in moderate DA [16].

NEW patients’ median disease activity according to the DAS28 before starting bDMARDs is somewhat lower in BIOREG compared to other European bDMARD registries (4.13 compared to 4.2–6.6), while median HAQ-DI scores are in a comparable range (1.0 vs 0.8–1.9) [14]. bDMARDs in RA in Austria are restricted to patients having failed to thrive on at least one csDMARD (preferably MTX), and only rheumatologists are licensed for their prescription [17]. Although this may suggest a more liberal prescription of bDMARDs in Austria than in other European countries, initiation of bDMARDs in Austria follows the relevant EULAR recommendations [18].

Starting bDMARDs in moderately active diseased patients showed somewhat better numerical outcomes as measured by different instruments than commencing at a high disease activity level [16, 19]. Regarding all patients after 12 months since BIOREG inclusion (*n* = 511), the median DAS28 was at the upper limit of remission (2.60 (25th and 75th percentile 1.80; 3.45)) and the median RADAI-5 was in the mild disease activity range (2.2 (1.0; 3.6)), indicating efficacy when assessed by a composite index and by a patient-reported outcome. This result may also be seen as an indicator that the patients included into this observation may be representative for all RA patients on the register.

As expected, in more NEW patients AEs were recognized during their first year of treatment with bDMARDs than in EST patients. Those AEs were mostly injection site reactions. Although NEW patients show higher disease activity, more frequently receive a higher amount of GC and csDMARDs and have more concomitant diseases, such as cardiovascular diseases, compared to EST patients, no more infectious complications were observed than in the EST group [20]. This finding may be in part due to the relatively short median exposure to bDMARDs in the EST group, namely 3 years, compared to an observation in the German register RABBIT, where 3 years was the cut-off to be regarded as “new on the drug” [21]. Compared with the RABBIT register, on which 13 % of the included patients experienced an infection in the first year of observation (out of them 6 % received a csDMARD without bDMARD), a similar overall percentage of infection of 11.7 % in all available AEs reports after 1 year in BIOREG is reported [22]. However, there remains a discrepancy in the valuation of seriousness: whereas in BIOREG 53 % of these infections are indicated as serious in all available AE reports (54 % in EST, 25 % in NEW), in the RABBIT register this percentage is 26 % [22], which may be founded on different therapeutic strategies concerning infections. NEW patients fulfil the criterion in the RABBIT register, where patients are included at the start of bDMARD therapy, whereas EST patients in BIOREG are on bDMARD therapy for a median 3.2 years. Since most reports about this issue conclude that serious infections are most likely during the first years of bDMARD therapy and are associated with age, co-morbidities, concomitant GC use, high-dose bDMARD, previous treatment with csDMARD and disability [23–26], further evaluations of this finding seem necessary to elucidate this discrepancy.

The most striking differences between the EST and NEW patient groups were related to concomitant treatment. EST patients took GCs (approximately 25 %) and csDMARDs (almost 50 %) much less frequently than NEW patients at the beginning, as well as after 1 year. This also mirrors the general intention to reduce the amount of medication in cases of successful treatment with bDMARDs. The overall use of GCs in the biological era is decreasing, although it is still high in clinical practice, at about 50 % on average [27]. Compared to more than 70 % GC co-treated patients in the German RABBIT register, concomitant GC use in NEW BIOREG patients was found to be comparable, namely 66 % at baseline [28]. GCs are an important factor for co-morbidities in RA patients [29, 30], not only for infections but also for conditions such as osteoporosis, diabetes, cardiovascular diseases or skin atrophy. A fortiori, around 25 % of GC users in the EST patients give hope that the use of GCs may be reduced in the future and underlines the effectiveness of bDMARD therapy.

At baseline, 44 % of the EST patients and 16 % of NEW patients received their bDMARD in monotherapy. In line with this finding, csDMARD therapy was stopped during the 1-year observation in 4 % of the EST patients and in 16 % of the NEW patients. An evaluation of all RA patients included in BIOREG revealed approximately 40 % of patients receiving bDMARDs as monotherapy at baseline. The percentage of RA patients on bDMARD monotherapy seems to increase with time of treatment [31], underlining the intention of Austrian rheumatologists to optimize therapy by reducing GCs and csDMARDs and keeping the patient on the ultimately effective drug after having achieved the treatment goal. In the case of treatment success, Austrian rheumatologists follow the EULAR recommendations for adopting therapy when the treatment target is reached [18]. bDMARD monotherapy seems not to be exceptional in Europe, even though lower rates are commonly reported (19–27 %) [32, 33].

In patients with two or more csDMARD failures, the addition of a bDMARD doubles the chance of achieving remission compared with switching to a csDMARD [16]. In the report from the RABBIT register, high remission rates (DAS28 remission: 30.6 %; ARA remission: 16.9 %) were observed in bDMARD patients with moderate disease activity according to the DAS28 at the start of treatment. These rates decreased to 8.5 % in patients with DAS28 > 6 at the time point of starting bDMARD [16]. These findings underline those derived from BIOREG. Not adding a csDMARD as a double- or triple-therapy, which has also been shown to be clinically effective [34, 35] with no difference in loss of work [36] but higher drop-out rates in the combination csDMARD groups, is an expensive approach [37]. In BIOREG, only 2 % of EST and NEW patients were on a combination of csDMARDs when starting the bDMARD. A 1-year course of therapy with a bDMARD in Austria costs €14,300– €20,000, depending upon the drug and the dosage and administration [38]. Austria may be classed among countries with a liberal prescription policy [39]. However, a Treat to Target principle, with consecutive bDMARD after two csDMARD failures, seems to be cost effective after 2 years of treatment [40]. In addition, it should be considered that patients in BIOREG were treated for a median 6 years in EST and 4 years in NEW patients with several csDMARDs. Since bDMARDs are expensive and carry risks of adverse events, trials have been conducted with stopping TNFα-blockers [41] or tapering them down when remission or stable low disease activity has been achieved [42]. Decanting TNFα-blockers shows worse clinical outcome than continuing, whereas cautious dose reduction at least for Etanercept or Adalimumab could be regarded a valuable alternative.

This study has limitations, the most important being the small number of NEW patients, as a result of the intention to include only patients with a full dataset available for a longitudinal study on tolerability and disease activity and therefore to avoid missing data [43]. Since BIOREG includes patients irrespective of the date of their initiation on bDMARDs, this disparity is an unavoidable drawback. Therefore, the presented data may give insights into the use of bDMARDs in Austria, but the section referring to the seriousness of AEs in particular has to be regarded with caution.

## Conclusion

In summary, this first evaluation of data extracted from BIOREG, the register for bDMARDs in Austria, indicates successful RA treatment with bDMARDs in Austria at reasonable tolerability. Validity of the data could be evidenced by showing NEW patients at comparable disease activity as EST patients after 1 year of bDMARD therapy. Plausibility is indicated by comparison of the results with the RABBIT register, with one exception, namely the high rate of serious AEs in the form of infections in EST patients. Starting bDMARD at lower disease activity than in other European countries is possibly accompanied by higher remission rates, not only expressed by DAS28 or BC but also indicated by a patient-reported outcome, the RADAI-5, and higher rates of bDMARD monotherapy. This fact may imply more cost-intensive treatment while suggesting the possibility of better outcomes for patients. Additionally, after having achieved the treatment goal, it appears to be general practice in Austria to primarily reduce or even stop treatment with csDMARDs and GCs, while keeping patients on bDMARDs to maintain the therapeutic success.

## Abbreviations

ACPA, Anti Citrullinated Peptide Antibodies; AE, adverse event; BC, Boolean remission criteria according to the 2011 ACR/EULAR remission criteria; CRF, Case report form; DA, disease activity; bDMARD, biological DMARD; csDMARD, concomitant conventional synthetic DMARD; DAS28, Disease Activity Score using ESR out of 28 joints; ESR, erythrocyte sedimentation rate; EST, patients with established bDMARD treatment before inclusion in BIOREG, for whom a full data set as described is viable; GC, concomitant glucocorticoid treatment; HAQ-DI, Health Assessment Questionnaire Disability Index; LEF, Leflunomide; MTX, methotrexate; N, number of patients; NA, not applicable; NEW, patients included in BIOREG with start of biological treatment ± 30 days, for whom a full data set as described is viable; RA, rheumatoid arthritis; RADAI-5, Rheumatoid Arthritis Disease Activity Index-5; RF, Rheumatoid Factor; SSZ, Sulphasalazine; SJ, swollen joint; TJ, tender joint; VASGH, patient’s global assessment of the disease on a 100 mm visual analogue scale; VASPH, physician’s global assessment of disease activity on a 100 mm visual analogue scale
