# Erratum to: Validity of data collected in BIOREG, the Austrian register for biological treatment in rheumatology: current practice of bDMARD therapy in rheumatoid arthritis in Austria

**DOI:** 10.1186/s12891-016-1270-x

**Published:** 2016-10-03

**Authors:** Bernhard Rintelen, Jochen Zwerina, Manfred Herold, Franz Singer, Johann Hitzelhammer, Wolfgang Halder, Gabriela Eichbauer-Sturm, Rudolf Puchner, Miriam Stetter, Burkhard F. Leeb, on behalf of the BIOREG investigator group

**Affiliations:** 1 Lower Austrian State Hospital Stockerau, 2nd Department for internal medicine, Lower Austrian Center for Rheumatology, Landstrasse 18, Stockerau, 2000 Austria; 2 Karl Landsteiner Institute for Clinical Rheumatology, Landstrasse 18, Stockerau, 2000 Austria; 3 Ludwig Boltzmann Institute of Osteology at the Hanusch Hospital of WGKK and AUVA Trauma Centre Meidling, 1st Medical Department, Hanusch Hospital Vienna, Heinrich-Collinstrasse 30, Vienna, 1140 Austria; 4 Medical University of Innsbruck, Anichstrasse 35, Innsbruck, 6020 Austria; 5 BIOREG, Schloßhoferstrasse 4/4/12, Vienna, 1221 Austria; 6 Health Center Vienna Mariahilf, Wiener Gebietskrankenkasse, Mariahilfer Strasse 85-87, Vienna, 1060 Austria; 7 Tyrolean State Hospital Hochzirl, Hochzirl, 6170 Austria; 8 Office based rheumatologist, Freistätter Strasse 16, Linz, 4040 Austria; 9 Office based rheumatologist, Freiung 19, Wels, 4070 Austria; 10 Department for Internal Medicine, Lower Austrian State Hospital Amstetten, Krankenhausstrasse 21, Amstetten, 3300 Austria; 11 Medical University of Graz, Auenbruggerplatz 2, Graz, 8010 Austria

## Erratum

After publication of the original article [1], it came to the authors’ attention that in the third paragraph of the ‘Discussion’ section, the value of DAS28 in NEW included patients is indicated wrongly, although correctly cited in Table 2. The correct form of the erroneous sentence is as follows:

“NEW patients´ median disease activity according to the DAS28 before starting bDMARDs is in the lower range in BIOREG compared to other European bDMARD registries (4.46 compared to 4.2–6.6), while median HAQ-DI scores are in a comparable range (1.0 vs 0.8–1.9).”

To correct the original sentence, “(4.46 compared to 4.2–6.6)” should be updated to “(4.13 compared to 4.2–6.6)”, and “in the lower range” should replace “somewhat lower”.

## References

[1] Rintelen B, Zwerina J, Herold M, Singer F, Hitzelhammer J, Halder W (2016). Validity of data collected in BIOREG, the Austrian register for biological treatment in rheumatology: current practice of bDMARD therapy in rheumatoid arthritis in Austria. BMC Musculoskelet Disord..

